# Psychological Stress and Cellular Aging in Cancer: A Meta-Analysis

**DOI:** 10.1155/2019/1270397

**Published:** 2019-11-13

**Authors:** Joanna Kruk, Basil Hassan Aboul-Enein, Joshua Bernstein, Magdalena Gronostaj

**Affiliations:** ^1^Faculty of Physical Culture and Health, University of Szczecin, Piastów 40b/6, 71-004 Szczecin, Poland; ^2^Faculty of Public Health & Policy, London School of Hygiene & Tropical Medicine, 15-17 Tavistock Place, London WC1H 9SH, UK; ^3^College of Graduate Health Studies, A.T. Still University of Health Sciences, 800 W. Jefferson St., Kirksville, MO 63501, USA; ^4^Faculty of Medicine, Biotechnology and Laboratory Medicine, Pomeranian Medical University, Rybacka 1, 70-204 Szczecin, Poland

## Abstract

**Background:**

Epidemiological evidence continues to accumulate on the effect of psychosocial and behavioral factors in relation to cancer risk, progression, and mortality.

**Material and Methods:**

This article presents the current evidence on the relationship between psychological stress and the risk of cancer and cellular aging process. Ten databases were searched to identify publications up to September 2019. References from retrieved articles were also reviewed. We included nine review papers and 26 cohort or case-control studies based on inclusion/exclusion criteria.

**Results:**

Results of previously published review articles did not show consistent evidence for the association between cancer risk and psychological stress, while previous evidence is stronger regarding the role of chronic psychological stress on cancer growth and metastasis and aging. In seven observational studies, severe life events, anxiety, depression, insufficient social support perception, or avoiding coping strategy were significantly associated with breast cancer risk. For other specific types of cancer, 11 studies reported increased risk factors for stressful life events, and two others found increased mortality or a decline in treatment adherence.

**Conclusions:**

Recent epidemiological evidence generally suggests psychosocial factors may be considered risk factors for specific types of cancer and play a key role in the cellular aging process. Understanding molecular mechanisms of the stress interaction is important in cancer management and prevention. The psychological stressors should be considered when developing or evaluating change in psychosocial practice.

## 1. Introduction

The premise that stress-related psychological factors influence the development or progression of cancer dates back 20 to 30 years of contemporary research [1–3]. Over time, the association has been widely discussed in the literature among various professional fields. Despite an extensive period of research, the literature findings are often dispersed between the fields [2] largely due to the complexity and multifactorial etiology of cancer, psychological stress (PS), and stress-related diseases [4–6]. In vitro studies on animals show PS can affect all three stages of carcinogenesis. In humans, PS influences the main processes in cancer pathogenesis such as DNA repair, cellular aging, alternations in the immune system, and apoptosis [7, 8]. Cancer is among the leading causes of death globally with 8.2 million deaths in 2012 [9] and 18.1 million new cancer cases and 9.6 million cancer deaths in 2018 for 36 types of cancers with lung, breast, and colorectal cancers as the most common types [10]. Evidence suggests 5-10% of all cancer risk factors have a genetic predisposition, and approximately 40-45% are determined by physiology, lifestyle (e.g., diet, physical activity, smoking, and drinking), and environmental risk factors [11]. Up to 20% of the cancer burden is associated with obesity. Evidence shows that 33% of lung cancers, 42% of breast cancer (BC), 43% of colon cancer, and 20% of prostate cancers are preventable through healthy lifestyle habits and preventive screening [12]. Psychosocial factors (e.g., mental stress, adverse life events, long-term depression, and social isolation) can adversely influence energy balance which contributes to the development of obesity [13].

The level of biological processes affected by PS depends on its duration [14]. Short-lasting PS activates the sympathetic nervous system (SNS) secreting catecholamines (CATs), which may exert beneficial effects [15]. In contrast, long-lasting persistent PS or high levels of PS are accompanied by biological, psychological, and behavioral changes and may have adverse consequences on health. Recently, there is growing evidence that depression is accompanied by increased levels of proinflammatory cytokines [16] and is hypothesized as a risk factor for cancer incidence and survival rates. There is ongoing debate on whether psychosocial factors should be considered risk factors for cancer development; until recently, the results are sparse and ambiguous. Due to increasing prevalence of cancer disease incidence and mortality as well as many sources which generate PS, an understanding the strength of the PS cancer association is important for the public health.

To our knowledge, the recently published meta-analyses evaluated the observational findings published up to 2017 [16, 17]. Since this time, several new studies have appeared. In this overview, we present the evidence on the relationship between PS, depression, and cancer and important findings from selected previously conducted reviews that synthesized this evidence based on observational studies published earlier. We also present the proposed biological mechanisms linking PS to the onset and progression of cancer and cellular aging, emphasizing the possible important role of oxidative stress (OS). We also identify gaps of the observational studies to provide a more complete picture of the state of knowledge in this area of research.

## 2. Materials and Methods

### 2.1. Search Strategy

Peer-reviewed research articles were identified by applying search strategies using databases: PubMed, Scopus, ScienceDirect, SpringerLink, Wiley Online, Taylor & Francis, ArticleFirst, ProQuest, PsycINFO, and EBSCOhost. A combination of search terms and key words included Psychological stress (self-reporting stress, psychosocial stress, major life events, domestic violence, depression, mental disorders) and cancer or tumor or carcinoma and outcomes (risk, incidence, mortality) and their combination. The databases were chosen due to their extensive coverage of cross-disciplinary and biomedical research scope and objectives. In addition, we hand-searched and cross-tabulated the reference lists of relevant articles, reviews, and meta-analysis papers. A comprehensive database search was finalized in September 2019. We limited the search to literature published in English.

### 2.2. Inclusion and Exclusion Criteria

This review included only the most recent articles reporting observational epidemiologic studies, systematic reviews, meta-analyses, cohort studies, and case-control studies that provide new information on the association between PS among different types of cancer survivors. Other article types such as conference abstracts, short communications, commentaries, editorials, brief reports, position papers, and hypothesis-generating statements were excluded based on lack of scientific merit. Only studies published in peer-reviewed journals were included. Inclusion criteria for this paper were physician-diagnosed cancer, given PS measurement tools, provided information on cancer type and association between PS and cancer type or overall cancers. We included case-control studies when odds ratio (OR) with 95% confidence interval (CI) or *P* value for statistical significance and numbers of cases and controls for each specific cancer site and type of psychosocial factors measured and matched or adjusted for age. Cohort studies were included if they reported relative risk (RR) or hazard ratio (HR) estimates and incident cases and subject (person-year) or they reported the number of cases and controls, and risk estimates were adjusted for confounding.

### 2.3. Study Selection

We selected original human studies: case-control studies, hospital-based case-control studies, prospective cohort studies, and prospective cross-sectional studies, which reported estimates of the relationship between self-reported psychosocial stressors, depression, and cancer risk. Outcomes included cancer risk for the following specific cancer sites: breast, brain, pancreas, colon, rectum, stomach, prostate, lung, cervical, bladder, the central nervous system, and white blood system (leukemia).

When considering multiple articles on the same population, the article based on longer follow-up intervals and contained more data was selected for consideration. Finally, 35 studies (26 observational studies and 9 review articles) were included in this review.

### 2.4. Data Extraction

Two research staff members (J.B. and M.G.) independently screened the titles and abstracts and evaluated the full-text articles. In the case of any disagreements regarding article inclusion, the problem was resolved through discussion with a third member of research staff (J.K.). Details on the type of study, study design, authors, publication journal and year, country where the study was carried out, study design, participant characteristics, specific outcomes, components of PS assessment and measurement tool, subtype of cancer, number of cases, number of controls, follow-up period, effect size, estimates of relationships and their measure with 95% CI or *P* value, variables of adjustment, statistical methods, and discussion of the study limitation were extracted. Only those articles reporting or assessing risk for cancer disease associated with PS and depression and reporting effect size and 95% CI based on sufficiently large samples were included in this review. Both case-control and cohort studies included in this paper showed key elements of study design, provided eligibility criteria, clearly defined outcomes (type or subtype of cancer and components of PS), had the representative numbers of cases and controls, and reported effect size adjusted for potential confounders.

## 3. Results

**Figure 1:**
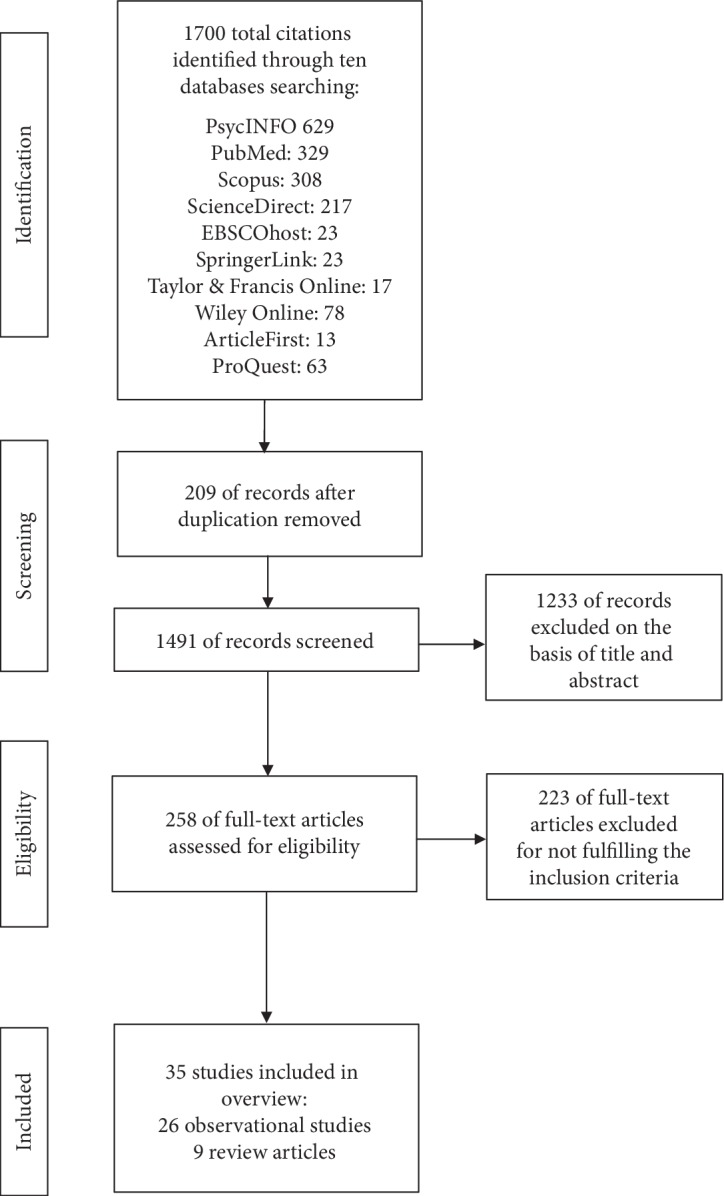
Flow diagram of literature search process.

During the search of ten electronic databases (Figure 1), a total 1,700 titles were identified.

After screening the titles and abstracts, 1,233 studies were excluded on association merit, 209 were excluded as duplicate studies, and 223 were excluded after review as the data did not meet inclusion criteria. Thus, observational studies—14 cohort studies and 12 case control studies linking PS with cancer—were analyzed in this review. In addition, the findings of nine review and meta-analysis studies were discussed. The studies were performed in different continents: America, Asia, and Europe.

### 3.1. Reviews and Meta-analysis Finding

Epidemiological research on this topic was a subject of a number of reviews, including those recently published by Chida et al. [18], Schraub et al. [19], Moreno-Smith et al. [20], Antonova et al. [1], Heikkilä et al. [21], Denaro et al. [2], Jia et al. [16], Chirac et al. [22], and Yang et al. [17] which reviewed studies published between 1940 and 2017.

The previous meta-analysis study by Chida et al. [18] investigated the association between stress-related psychosocial factors and cancer risk. The authors found PS was significantly associated with increased lung cancer incidence among initially healthy individuals; shortened survival time in patients with breast, lung, head and neck, hepatobiliary, and lymphoid cancers; and higher cancer mortality; however, the magnitudes of HRs appeared to be very small. Although the authors' findings were based on a large number of the observational studies (165) and calculated HRs were statistically significant, the meta-analysis study combined data from studies with different variables (e.g., clinical outcome, treatment, population, measurement methods, control for confounding for lifestyle factors for a particular subtype of cancer, and what can influence effect estimates). Thus, the study provided evidence of a low positive association between stress-related psychosocial factors and cancer.

Schraub et al. [19] reviewed 32 studies on the relationship between life-event stress, depression, and the risk of several types of cancers mainly BC, published between 1940 and 2004. The included eighteen studies showed no association between PS factors and cancer risk, and six studies found a significant association in one or several subgroups. The authors estimated PS may increase BC risk, although four of nine studies presented no significant increase, and one study showed a decrease in risk. Majority of the 11 studies noted no significant association between stressful life events and overall cancer risk. For specific cancers (leukemia, lymphoma, melanoma, colon, and cervix), more than a 20% increase in cancer risk was reported. In addition, a 65% increase in the risk for tobacco-related cancers and significantly decreased risk of endometrium cancer (33%) in users of hormone replacement therapy (HRT) and among normal weight women (37%) were noted. Also, a 40% decreased risk of colorectal cancer occurred within the female subgroup for moderate intensity of PS. Findings were controversial for relationships between depression or personality and risk for cancer. The authors found a 38% increase in cancer incidence and 20-96% increase in mortality among individuals suffering from depression (in two of seven studies) and a 20% decrease in the risk (in one study). Only one of the six studies presented a significant increase in cancer risk when personality was a factor. It is noteworthy that Schraub et al. [19] analyzed only cohort studies and case-control in population cohort, but not the classical case-control studies to minimalize the influence of selection bias and recall bias. The authors suggested no conclusions regarding life events or depression and cancer development or progression can be drawn.

Antonova et al. [1] reviewed 16 studies published between 2000 and 2010 focusing on associations between different types of stress-related events (work-related stressors, daily stress, and war- and conflict-related exposures) and BC incidence. The authors noticed inconsistencies in the findings, e.g., a strong causal link between high job demand, job strain, or severe life events (divorce, separation, or death of husband, child, or a close relative) and BC risk (HR ranged between 1.12 (1.0-1.25) and 2.65 (1.06-6.60)) reported in several studies and lower risk associated with high intensity stress HR: 0.60 (0.37-0.97) shown in other studies. The authors concluded the stress hormone cortisol may increase the rate of BC growth and the PS-cancer risk association was strongly dependent on the type of stress and on stress timing, particularly, for the stress induced by major life events, e.g., maternal death in childhood.

A meta-analysis performed by Heikkilä et al. [21] of data from 12 prospective European cohort studies (116,056 individuals aged 17-70 free from cancer at baseline with a follow-up period 1985-2008; 5,765 all cancer cases: 522 colorectal, 374 lung, 1,010 BC, and 865 prostate cancer (PCa) identified) on work stress and high job strain reported multivariable adjusted HR: 0.97 (0.90-1.04) for overall risk of cancer and also nonsignificant risks for the specific cancers.

Denaro et al. [2] reviewed seven observational studies published between 1995 and 2009, including one meta-analysis study. The findings of the meta-analysis did not confirm an overall relationship between PS-related life events and BC risk, although the data reported a modest correlation for death of spouse. Four reviewed studies observed a positive correlation between life events and BC, HR, or OR ranged from 1.12 (1.0-1.25) to 3.70 (2.61-5.26), but two studies were not controlled for confounding factors. The authors concluded epidemiologic evidence provides a strong support for a positive correlation between PS and BC risk, but variables determining the stress-cancer link were numerous and difficult to verify.

Further, the recently published systemic review by Chirac et al. [22] including all studies from 1966 to 2016 (52 eligible studies) on the effect of PS on BC found positive associations between experience of stressful events, personal traits, and BC in 26 observational studies, negative correlations in 18 studies, and data in eight studies could not be classified. Their qualitative analysis suggested a possible association between stressful life events and cancer; however, they highlight the methodological heterogeneity within the studies.

In turn, Moreno-Smith et al. [20] reviewed the association between psychosocial factors, mainly chronic stress, and cancer progression, focusing on biological processes affected by chronic PS. The authors found strong evidence for the effect of chronic stress, depression, and social isolation on cancer progression and limited evidence for the role of behavioral factors in cancer initiation.

The recent systematic review and meta-analysis conducted on January 1, 2017 of 25 studies published during 1988-2015 by Jia et al. [16] on the association between depression and incident of risk for breast, colorectal, colon, liver, lung, and prostate cancers found depression significantly increased RR of overall cancers by 15%, liver cancer by 20%, and lung cancer by 33%. The authors noticed no significant association for BC, PCa, or colorectal/colon cancer and increased risk of overall cancer among North America individuals.

Another meta-analysis of observational studies of Yang et al. [17] on the association of work stress and cancer risk included nine studies (281,290 participants, 9,090 incident cases) published during 2004-2017. The authors found statistically significant effects of work stress on an increase of the risk of several types of cancer: colorectal, RR = 1.36 (1.16-1.59); lung, RR = 1.24 (1.02-1.49); esophagus, RR = 2.12 (1.30-3.47), but not on prostate, breast, or ovarian cancers. However, the authors found the effect of work stress on colorectal cancer risk was significant only among participants from North America and increased risk was high (50%), but the association was not significant among participants from Europe. On the contrary, the effect of work stress on esophagus cancer risk was found to be significant in Europe, but not in North America.

### 3.2. Observational Studies

A summary of the evidence relating PS to BC risk from observational studies published during 2011-2018 is shown in Table 1.

A hospital-based case-control study by Kruk [23] included a large sample size of women with histopathological confirmation of the cancer (*n* = 1,943). Participants were characterized by detailed information on potential confounders using a self-administrated structured questionnaire, and the risks of BC were estimated in multivariate analysis and tests for linear dose response was conducted. Several major life events, like death of a close family member, personal injury, illness, troubles with the law, or retirement, were statistically significant in their association with BC risk; the increased risk ranged from 1.58 to 2.94. Additional analysis showed significantly increased risks for two periods of PS events—lifetime and beginning at birth—and ending 5 years before the cancer diagnosis, for life score levels > 70, being the highest for scores > 210. Life events' scores were estimated based on the Holmes and Rahe social readjustment rating scale [33]. The highest mean weight on this scale is 100 scores (death of husband); divorce was scored at 73 points, separation at 65 points, and death of a close family member at 63 points.

Wang et al.'s [24] case-control study identified the association of PS alone and combined lifestyle determinants are considered potential risk factors (low physical activity, alcohol intake, cigarette smoking, diet rich in animal meat, and high intake of fried food) with BC risk. After adjusting for known risk factors, the authors noted a 65% increase in risk for high perceived PS; ORs ranged from 1.89 to 3.36 when perceived stress was combined with these risky lifestyle behaviors.

Li et al. [25] used a comparative case-control study of 582 women with benign BC and 540 controls, aged ≤40 years showing that frequent depression, negative emotion (e.g., fear, worries, nervousness, sorrow, and helplessness), and disharmonious marital status were associated with the development of early onset BC.

Sawada et al. [26] analyzed data from 29,098 women from 23 study areas throughout Japan to find the relationship between BC incidence and psychological traits. PS was evaluated using subjects' response to questions: having “ikigai”, i.e., “something that makes one's life worth living”, decisiveness, ease of anger, and perceived stress of daily life with 3- or 4-point Likert-type response (disagree, neither, agree, and agree strongly). They found that none of the psychological traits were significantly associated with BC risk. However, this study has important limitations noted by the authors, i.e., 1-item measures of stress which could attenuate the true relationship between BC incidence and each item.

In turn, a prospective cohort study by Schoemaker et al. [27] comprising a large sample of women aged ≥16 and focusing on BC etiology used a postal questionnaire of which a follow up was repeated every 2.5-3 years to update risk factor information and obtain data on BC diagnosis. Researchers identified 1,510 cases with invasive cancer and 273 with in situ cancer during an average follow-up period of 6.1 years. The study tested a wide range of PS variables and carried out analyses by estrogen-receptor status, including BC risk factors (physical activity, obesity, alcohol intake, and smoking). This study observed no association between adverse life events, evaluated as the highest score in the Holmes and Rahe scale, and cancer, except a 31% increased BC risk among a subgroup of women under 20 years who experienced maternal death. However, the risk was not elevated when their mother had BC or ovarian cancer.

Yeh and Lee [28] analyzed data from a medical center's outpatient department in Taiwan whose patients were scheduled to receive mammography screening. The authors grouped participants into cases and controls based on pathological biopsy results. The Perceived Stress Scale measured general, life, and work-related stress perception. The Hospital Anxiety and Depression Screens for symptoms of anxiety and depression combined were applied. Participants completed demographic, lifestyle, and medical history basic characteristic questionnaires. The results showed participants with borderline anxiety were approximately 3 times more likely to have BC compared to subjects with no anxiety; subjects with depression had 4.5 times higher BC risk than subjects with no depression. The authors estimated that every point added to the average total stress score increased BC risk by 1.124 times concluding that stress, anxiety, and depression may be predictors of BC risk.

The Özkan et al.'s [29] case-control study analyzed the association between PS and social support with BC risk using the Stress Assessment Form and the Coping Strategy Indicator. The first form included data on childhood trauma (e.g., death of mother, death of father, and divorced parents) and major life events (e.g., death of husband, divorce, and a chronic serious disease). The second form contained 33 items for an evaluation of brief coping inventory. The results showed the increase in BC risk may be related to general life stress, such as existence of a stressor experience, perception of inadequate social support, or use of avoidance social support coping strategies.

The recent prospective cohort study by Butow et al. [30] was comprised of Australasian women aged 18-75 on the relationship between life-event stressors, social support, personality, and risk of developing BC among women with history of increased familial of BC. The authors applied semistructured phone interview, the Life Events and Difficulties Schedule interview protocol, and other questionnaires to estimate social support, optimism, and antiemotionality to obtain data on acute or chronic severity, e.g., death of a spouse/child, handicapped child requiring full-time care, and moderate stress such as buying/selling a home. The authors also identified eight psychosocial variables as predictors or moderators of the effect of stress on BC development, e.g., social support, personality, acute chronic stressors, and optimism. The authors did not report significant associations between any life-event stressors independently on its intensity in unadjusted and adjusted models.

Fisher et al.'s [31] case-control study of women aged 24-75 years identified evidence for life events perceived as stressful (abortion, illness, and relocation) and nonstressful (death of sibling, illness, and illness in family) considered as potential risk factors for BC. After adjustment for known risk factors for BC development, the authors found a cumulation of adverse life events perceived as stressful was significantly linked with increased risk for BC in a dose-response manner (OR = 1.63 (1.00-2.66), *P* trend = 0.045) and those life events perceived as nonstressful did not show statistical significance in the PS-cancer relationship. Further, regardless of personal illness perception (stressful or nonstressful), previous illness increased BC risk (OR = 2.84 (1.96-4.11), OR = 3.47 (1.34-8.94), respectively). Also, regardless menopausal status, nulliparous women or who had their first child at ≥30 years of age had increased BC risk.

Yildirim et al. [32] used a hospital-based case-control study of 250 cases treated for BC (surgery, chemotherapy, and radiotherapy), aged 27-64 years, to estimate PS influence on BC risk, applying semistructured interview, the Stress Evaluation Form, and the Healthy Lifestyle Behavior Scale. Lifelong stressful events such as childhood trauma (e.g., loss of mother or father, divorce of parents, or serious health problems), major life events (death of a loved one, serious disease, and divorce), chronic stressor (e.g., interpersonal relationship problems), and experience of stress within the last five years before disease were collected. The authors found that loss of father during childhood increased BC risk 2.68 times, inadequate social support 1.83 times, and serious stressor within the last five years 4.72 times. Also, psychiatric history was a factor increasing BC risk (1.95-fold). The authors underlined the particularly important influence of stressful events within the last five years on BC development.

A summary of the evidence linking PS to cancer other than BC is given in Table 2.

A hospital-based case-control study by Cabaniols et al. [34] included patients aged 18 and older with previously untreated glioma grades II-IV. The authors found a 90% significant increase in the risk of malignant primitive brain cancer caused by major PS life events and insignificant decreased risks related to daily stress OR: 0.90 (0.49-1.71) and to stress at work OR: 0.69 (0.27-1.74), measured over five years before cancer diagnosis. The authors suggest genetic factors are involved in glioma cancer and unexpected acute PS may participate in malignant primitive brain tumor development.

The Huang et al. [35] study was a large nested case-control study that included the Swedish population and health registers, which examined whether the death of a child was linked with pancreatic cancer in men and women aged 55 years and older. The authors noted a 9% increase in the risk associated with the death of child (overall). The risk increment to 27% was observed within the first five years after a child's death and also for loss of a child due to suicide (23%). The PS-pancreatic cancer association was only statistically significant in women (a 37% increase in the risk compared to controls) and in men with psychiatric illness, OR = 2.1 (1.52-2.91). In addition, the authors found participants with a history of psychiatric disease experienced increased pancreatic cancer risk to the greatest extent after child loss.

The Vasunilashorn et al. [36] study included a cohort of Taiwanese adults aged above 53 years and characterized the association between perceived stress based on six items dealing with the respondents' and their family health, financial situation and occupation, and mortality risk during the 11-year follow-up period. The authors found perceived stress caused a 19% statistically significant increase in mortality risk only among individuals with poor health outcomes. The authors concluded the observed relationship between perceived stress and mortality may be dependent on a participant's current health.

The large follow-up study conducted by Momen et al. [37] used data from Danish and Swedish national registers studying the association between bereavement by the death of a close relative before 15 years of age as an indicator of severe PS and childhood leukemia cancer. The authors found an approximate 10% increase in the risk of all childhood cancers and a 14% increase for central nervous system tumors due to bereavement. They concluded that experience of PS in early life is linked with a small elevated risk of childhood cancer risk.

The Azizi and Esmaeili [38] case-control study conducted in four Iranian hospitals identified the relationship between stressful life events assessed using the Holmes and Rahe Life Events Questionnaire and colorectal cancer. After adjusting for known risk factors, the authors found 2.49 times higher risk of colorectal cancer linked with the death of loved ones compared with controls. Authors found stressful life events with lower weight on the Holmes and Rahe scale such as family disputes and job problems, and serious financial problems also increased risk, but without statistical significance. The authors suggest stressful life events may be a factor increasing risk of colorectal cancer.

Kikuchi et al. [39] analyzed data from 61,563 participants from the Japan Collaborative Cohort Study to measure the relationship between perceived stress estimated on a 4-point Likert scale and colorectal cancer incidence. The authors found a significant relationship between daily perceived stress of moderate or high/severe intensity and rectal cancer incidence, e.g., 2.16-fold and 1.75-fold increases in the risk among men, respectively, but not for colon cancer incidence. The higher HR for cancer incidence was observed in women at the moderate stress level than at the high/severe stress level, indicating a reverse U-shaped relationship. Due to wide 95% CI of the HRs rectal cancer and a lack of statistical significance for colon cancer, the authors recommend further research with a greater sample size.

The Blanc-Lapierre et al. [40] population-based case-control study analyzed the association between perceived workplace PS over the entire work career and cancer among men. The authors observed that employment in at least one stressful job (e.g., high demand, time pressure, financial issues, and job insecurity) was significantly linked with increased ORs of cancer at 5 of 11 sites, i.e., the lung, colon, bladder, rectal, and stomach with a duration trend. Nonsignificant increases in risk of cancer of the non-Hodgkin lymphoma, kidney, melanoma, pancreas, and esophagus were also noted. Short-term (<15 years) work-related stress was not linked with any cancer. Contrary, significant associations were noted for longer cumulative periods of exposure to perceived job stress (15-30 years or above 30 years) and the lung, colon, bladder, rectum, stomach, and non-Hodgkin lymphoma cancers, and borderline significant risk for prostate cancer. The authors recommend further studies with detailed assessment of all job stressors during employment carried out on larger case and control groups.

Kim et al. [41] examined the prevalence and prognostic significance of psychological distress (PD) in gastric cancer patients. The authors used three methods to measure the severity of anxiety, insomnia, and depression, and the degree of functional impairment. The data showed 33.6% of participants were identified as patients with PD. The patients with PD had worse disease-free survival rates compared with patients without PD. In stage IV of cancer, patients exhibited almost 2.5-times poorer overall survival rates than patients without PD, HR = 2.47 (1.07-5.68). The authors demonstrated that patients with gastric cancer, independent of cancer stage, experienced PD due to worse survival outcomes and recommend further studies on the role of psychotherapeutic intervention on improved patient survival outcomes.

A large cohort study by Chang et al. [42] on the role of depression in overall cancer and hormone-related cancer development in the general population (aged 30-60 years) observed differences in direction and magnitude among relationships in men and women for cervical cancer. Authors identified at baseline major depression in 7.4% men and 10.2% women. Major depression caused a 5% increase of total cancer in men and a 10% decrease in women. In turn, minor depression identified at baseline in 19.3% men and 21.4% women resulted in a 3% increase of the total cancer risk in men and was without effect among women. The authors suggested a lack of the association between depression and cervical cancer and BC development; however, a 13% increase of PCa risk in men affected by minor depression was observed. According to the authors suggestion, the depression-cancer association would benefit from future studies that consider specific cancer subtypes and mechanism interaction.

In turn, a large international study based on data from 19 countries by O'Neill et al. [43] reported the association between a number of retrospectively assessed lifetime prevalence of 16 DSM-IV mental disorders (anxiety disorders, mood disorders, substance use disorders, and impulse control), major depressive dysthymia, and risk of overall cancer. These authors found the positive association between a number of mental disorders (OR ranging from 1.3 to 2.3) and overall cancer risk; the magnitude of the relationship was higher in women, and the possible carcinogenic impact of mental disorders was different in various periods of respondent's life. Significant effects of depression were observed with cancer diagnosis in a subgroup of women up to age 44 and were strongly linked with cancers diagnosed early in respondents' life as well as in women. O'Neill et al. [43] maintain that early diagnosis and treatment of mental disorders is important to avoid the increased risk of cancer occurrence caused by inferior lifestyle characteristics.

The Archer et al. [44] prospective cohort study (Whitehall II, all London-based office staff) of 17.4 years follow-up time analyzed the relationship between chronic depressive symptoms and smoking-related (109 cases), hormone-related (311 cases), and other cancers (356 cases) using the General Health Questionnaire depression subscale among participants aged 35-55 years at baseline. The authors stated chronic depression was not associated with overall cancer incidence when estimated for significant years of follow-up time. However, the authors found significantly increased risk (89%) among patients who experienced a new episode of mental disorder in the first 9 years of follow-up time. In the authors' opinion, subclinical cancer diagnosis can directly affect the brain and elicit sickness behaviors and depressive symptoms.

Li et al. [45] carried out a case-control study nested in Shanghai city with 250 PCa cases and 500 controls, which examined whether psychosocial factors including occupation, marital separation, and suffering predisposed men to PCa risk. After adjustment for confounding hormone-related factors (lifestyle, eating habits), men who presented a high level of stress caused by negative psychosocial factors had significantly increased risk; RRs ranged from 1.61 for occupational factors to 2.37 for sensitivity to personal comments. Marital separation also exhibited a high magnitude of PCa risk increase (1.94-fold). In addition, the authors found decreased PCa risk with regular intake of green vegetables and green tea, increased risk with alcohol, red meat, and processed food consumption.

Song et al. [46] explored the association between perceived stress levels (baseline and updated after a 5-year follow-up period) and overall cancer risk (gastric, esophageal, colon, lung, prostate, breast, liver, rectal, and pancreatic). The authors used self-reported data from the Japan Public Center-based Prospective Study which enrolled 140,420 participants aged 40-69 years. Among them, 101,708 individuals declared perceived stress. Increased risk for overall cancers by 11% was observed among all participants that experienced consistent high intensity PS (*P* − trend = 0.0002), by a 19% in men (*P* − trend = 0.0001) and by a 7% nonsignificant risk in women (*P* − trend = 0.1227) compared to those reporting constantly low stress levels. In separate analyses, cancer risk was significantly increased in men and was especially high among smokers, alcohol drinkers, obese, and those without family history of cancer. Using analysis by type of cancer, the authors reported the highest sensitivity to PS stress was observed for liver cancer (33%), PCa (28%), and pancreatic cancers (26%).

Using a large case-control study, Blanc-Lapierre et al. [47] analyzed the association between perceived lifetime workplace stress and newly diagnosed PCa risk. Individuals answered questions to determine whether their job made them feel tense, anxious, or stressful most of the time. There was a dose-response increased PCa risk across stress duration categories. 58% of respondents recognized that at least one job was stressful during their professional career. The risk was increased among men aged ≤65 who reported more than 30 years of workplace stress by 12% per 10-year increment for both low-grade and high-grade PCa. The researchers concluded that PS was reported more often in white-collar jobs and was significantly linked to PCa diagnosed at a younger age.

Based on a large cohort sample (*n* = 6571) of cancer-free women from the Danish Nurse Cohort aged 45-70 years at inclusion, Vesterlund et al. [48] analyzed the association between prolonged job strain, measured using single items dealing work speed or load, the number of duties entrusted, and level of participants' ordinary effect on organization of duties during working time across six years and cancer risk (overall cancer, virus immune-related, hormone related, digestive, and the lung) based on self-reported questionnaires on job strain. The authors noted a high percentage of cancer cases with perceived PS, but no evidence of an increased risk of any cancer subtype among the examined group due to prolonged job strain. We suggest that too small numbers of respondents declaring high job-strain level (*n* = 692 compared with those declaring low job-strain level, *n* = 4,155) might also affect the results. Although, as the authors emphasize, the effect of job strain may differ between men and women and being dependent on several factors including lifestyle.

The Jafri et al. [49] study estimated the effect of major stressful life events on developing lung cancer using the Holmes and Rahe Life Event Questionnaire. The study was a case-control study matched for age, sex, and smoking status (but not for the duration of smoking exposure) with patients' median age of 64.1 years. The percentage of diabetes among controls was significantly higher than cases as well as diabetes and *β*-blockers users. The examined groups did not differ in a number of experienced stressors during lifetime, but there was significant difference in a number of major stressful events experienced in the preceding 5 years (higher among cases). The authors found an approximate 2.2-fold increase in the risk of lung cancer, due to stressful events experience within the preceding 5 years. Additionally, the authors observed that use of *β*-blockers may prevent against lung cancer development.

## 4. Discussion

This article summarizes the current information in a condensed form on behavioral factors, such as chronic PS, depression, mental disorders, exposure to job stress, and social isolation on the development and progression of cancer, and role of the stress in the cellular aging. We also provide current insights into some plausible biological mechanisms based on previously published reviews and meta-analyses and achievements in the last years. We also highlighted the complexity of this relationship and attempted to explain the contrasting findings between observational studies. The large number of epidemiological researchers reporting relationships between psychosocial and behavioral factors and cancer risk demonstrates the importance of PS in public health and cancer therapy. This update of the epidemiological evidence is based on analysis of nine previously published reviews of 225 worldwide findings and 26 cohort or case-control studies that did not show major biases a priori. Considering review articles for severe life events and BC, increased risk was concluded in five [1, 2, 17, 20, 22] of nine reviews. In addition, two of the nine reviews concluded a significant positive relationship between depression and social isolation and cancer progression [16, 20] although one review [34] reported controversial findings for depression where PS appeared to have a protective effect for colon and endometrial cancers. One review study [18] reported stress-related psychosocial factors could slightly shorten survival time in patients with diagnosis of BC as well as increase cancer mortality.

In the relationship between stressful life events and occurrence of other cancer types, the inconsistency of the epidemiologic studies reviewed and equivocal findings of a dose-response association lead us to conclude no significant relationship, with the exception of the positive association with cervix and colon cancers [21], colorectal (only in North America), esophagus and lung cancers [17, 18, 49] or endometrial and colorectal cancers, where stress occurred as a protective factor [19]. Two review papers highlighted the significance of chronic stress, social isolation, and depression on cancer progression, but limited evidence in cancer initiation [16, 20], and one review [19] maintained PS may shorten survival time in a few cancer subtype of patients. This issue might introduce selection bias because the relationship was weak; the authors do not rule out causal linkage.

Psychological stress at work is considered by the American Institute of Stress [50] as the most frequent type of stress. Job stress is characterized by high demands (“excessive workload and the need to work fast”) and degree of control (“low decision latitude”) [48]. Five of nine review papers studied associations between PS at work and overall cancer or specific types of cancer, and four reviews [1, 2, 21, 34] concluded a lack of statistically significant association or presented controversial findings. We identified main sources of observed inconsistencies in many articles being the subject of analyses including lack of an adjustment for fully established risk factors for particular types of cancer. For certain cancers, adjustment for lifestyle factors such as smoking, alcohol consumption, physical activity, body mass index, and family history of cancer disease in the regression models is essential for the proper evaluation of a risk.

We analyzed 26 observational cohort and case-control studies on different specific cancer sites and different psychosocial factors. We found authors analyzed associations between PS and cancer using different outcome measures, follow-up periods, countries, sample sizes, gender classifications, and controls for confounding factors. Of the 26 prospective cohort and case-control studies on the PS-cancer association, 10 studies included only data for BC (see Table 1). Seven of the 10 studies had a statistically significant risk increases when considering severe life events alone or combined with risky lifestyle behavior, anxiety, depression, insufficient social support perception, or avoidance strategy. The reported magnitude of the associations between stressors and BC were significant, e.g., OR = 1.76 for major events or OR = 5.66 for experience of several stressors [29]. Three of the 10 studies concluded severe life events [27, 30] or personality (e.g., decisiveness, perceived value of life) [26] do not play an important role in the BC etiology. Rather, the net sum of the evidence on the enhancing effect of severe life events on BC (seven of the 10 papers, 70%) remains in accordance with the findings of the abovementioned review papers.

Considering associations between stressful life events (death of loved ones, parents, spouse; daily life stress; marital separation; and self-containing suffering), depression, and specific types of cancer, all research articles noted increased risk factors, although one study [44] observed an increased risk among individuals with new depressive symptoms only and other authors [49] found an increase in the risk for stressful events in past 5 years (see Table 2). In addition, one study [36] reported increased mortality rates for self-perceived stress linked with poor health outcomes, financial troubles, occupational problems, or consequences of perceived stress for mortality, and the second study [41] found worse treatment adherence for all stages of gastric cancer in patients with psychological distress due to cancer diagnosis and treatment. These findings are in contrast to the reviews and meta-analyses analyzed in this overview that concluded no relationships. Regarding the impact of mental disorders and cancer risk and their interaction, the study by O'Neill et al. [43] reported an increased overall cancer risk at different life stages and its dependence on a number of disorders increased cancer risk even 2.3-times was found for more than five disorders; the magnitude of the association was also gender dependent. This study was based on self-reported diagnosis of cancer in 19 countries, including lifetime history of 16 different DSM disorders of each the effect was analyzed. However, their identification was based on respective recall as well as the data on cancer disease were self-reported. In addition, the risks were estimated for overall cancer but not for its specific subtypes. Thus, the relative strong positive relationships on the mental disorder-cancer outcome may suffer from bias. Notwithstanding, a large sample size and significant high magnitudes of the associations (1.3-2.3) suggest this study presents new information about the association between mind and cancer.

Six studies included separate risk estimates for the association between work place stress and specific types of cancer [25, 34, 40, 42, 47, 48], three of these studies found increased risk for prostate cancer [25, 42, 47] and one for lung, colon, bladder, rectal, and stomach cancers [40]. The remaining two studies found no effect of workplace stress on brain risk [34] and the hormone-related, virus immune-related, lung, and digestive cancers [48]. Thus, the significant cancer risk increases related to work were observed in 66.7% of retrieved observational studies.

Stress timing, time windows of stress exposure, stress type, methodological differences in the measurement of PS type, and individual's stress susceptibility are potentially important factors in a proper evaluation of the PS-cancer risk association. Variation in the applied scale to measure the same stressful life events presents as an important problem. Use of different PS test scales for a measure of subjective stress and subtype of cancer in the original papers, being the subject of this analysis, could lead to diversity and influence the magnitude and power of the relationships reported here. Continuing, this review shows most studies controlled for confounding factors, but only to some extent. An important issue when considering confounding factors is the role of the main risk factors for specific types of cancer, e.g., BC factors are linked with reproductive status, lifestyle, anthropometric characteristics, menopausal status, and ethnicity [51]. For example, body mass index and physical activity could attenuate risk estimates and influence on catecholamine levels in the blood, consequently on levels of O_2_^·−^ and other ROS/RNS toxic species [52]. However, it is important to note that in our review period, the majority of research studies examined specific cancer types other than BC.

**Figure 2:**
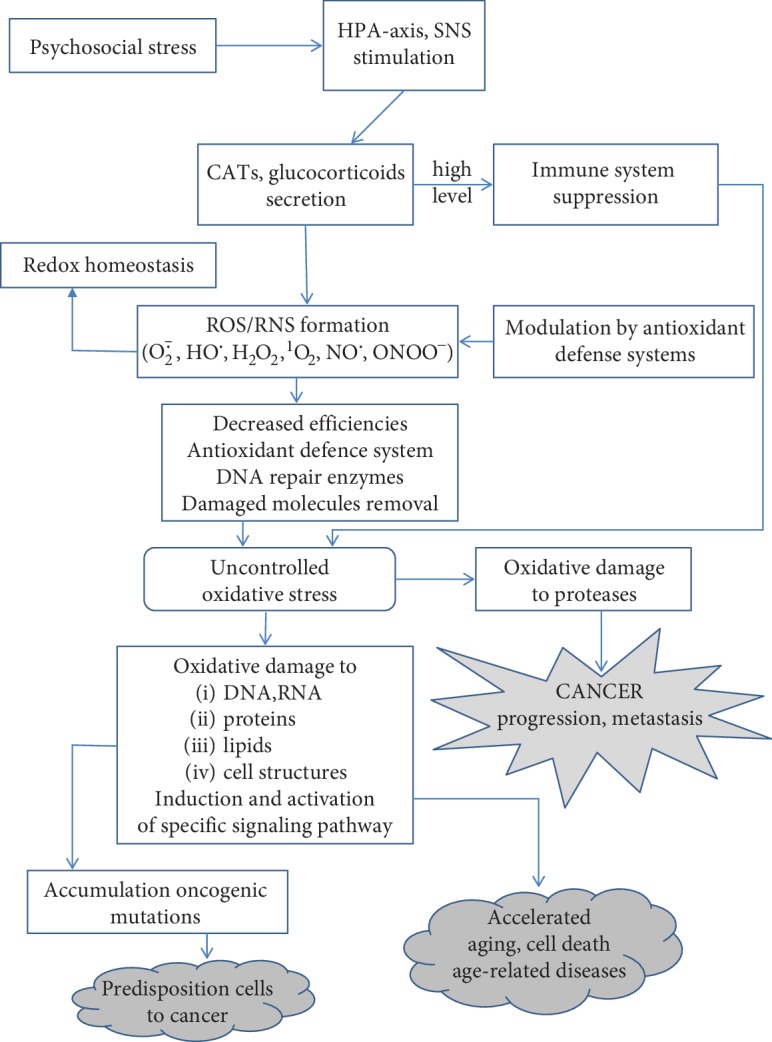
Simplified hypothetical scheme for the role of psychological stress in carcinogenesis and aging. CATs: catecholamines; ROS: reactive oxygen species; O_2_^·−^: superoxide anion radical; HO^·^: hydroxyl radical; H_2_O_2_: hydroxyl peroxide; ^1^O_2_: singlet oxygen; NO^·^: nitric oxide; ONOO^−^: peroxynitrite; HPA axis: hypothalamic pituitary adrenal axis; SNS: sympathetic nervous signaling.

Several biochemical processes are considered in the PS-cancer and PS-aging links including the activation of the hypothalamic pituitary adrenal (HPA) axis, deregulation of the sympathetic nervous signaling (SNS), inflammation, and decrease in cellular immunity [4–6, 8, 11, 20, 53–61] (Figure 2).

The HPA axis and SNS reportedly play an important role in all stages of cancer: initiation, growth, and progression [55]. During stress conditions, the HPA axis is activated, followed by signaling, generation, and release of adrenocorticotropic hormone which stimulates the adrenal gland to release CATs (adrenaline, noradrenaline). Also, other stress mediators like glucocorticoids (e.g., cortisol) participate in tumor growth and metastasis [6, 54, 56]. Cortisol arises from cholesterol and is released in response to stress and glucose from the adrenal gland acting as enhancers and suppressors of the immune system [56]. In turn, the SNS stimulates secretion of noradrenaline in the blood stream [54]. The excess CATs can affect cancer progression, regulating several cellular signaling pathways through adrenergic receptors (ADRs) of which expression was found in several cancer cells [6, 57]. ADRs act as enhancers of cancer cell proliferation and modulators of cancer cell interaction with their microenvironment to promote tumor progression. Under severe stress conditions, CATs activate *β*-adrenergic receptors on tumor cells and enhance expression of matrix metalloproteinases (MMPs) and vascular endothelial growth factor (VEGF) in adipose tissue, forming new blood vessels [55]. They also induce cell growth via promotion of cell cycle progression and prevention of apoptosis. The *β*-ADR stimulates adenylyl cyclase activity, an enzyme that catalyzes the conversion of ATP to cAMP, followed by protein kinase A (PKA) activation. The *β*-ADR/cAMP/PKA signal cascade can induce DNA damage or regulate expression of genes via activation of transcription factors [57, 59]. Also, elevated levels of CATs directly suppress the cell-mediated immunity acting as reducing agents of macrophages and T-helper lymphocytes (called Th cells) of cytokine producers (among other IL-12, TNF-*α*, and IFN-*γ*) and indirectly by stimulation of the immunosuppressive factor release, e.g., IL-10 and IGF-*α* [6, 56, 60]. Evidence has shown that cellular immune response during carcinogenesis is complex and multidirectional with anti- or proinflammatory activities depending on the tissue-specific microenvironmental stimuli [56]. There is strong evidence that chronic PS acts to suppress the natural killer (NK) cell activity and immune system power during cancer growth, progression, and metastasis [20, 62, 63]. The immune dysfunction accompanying PS, caused by the decreased production of antibodies, macrophages, monocytes, and T cells and inhibition of NK cells' activity play a key role in carcinogenesis. Evidence has shown that chronic inflammation occurs in several types of cancer. Morbidity and mortality were found to be correlated with proteins induced by PS such as IL-6 [3]. Also, immune cells such as cytotoxic T cells and NK cells were detected in invasive and metastatic tumors [20, 61]. Evidence on biological mechanisms potentially explaining increased cancer risk among individuals with depression suggests a direct effect on the immune system through the HPA axis as well as an indirect effect through unhealthy lifestyle, e.g., tobacco smoking, alcohol consumption, low physical activity—behaviors which are recognized as risk factors for some cancer subtypes [63, 64].

Alterations in key neurotransmitters were reported as important contributors to the increased level of reactive oxygen species (ROS) due to CATs' oxidation among other ROS sources [65]. As mentioned above, PS directly triggers the release of CATs and glucocorticoids, thus affecting the immune system. Activated inflammatory cells are known generators of ROS and reactive nitrogen species (RNS) involved in DNA damage and genomic instability [66–69].

ROS/RNS are generated in cells by biological processes (mitochondrial electron transport chain, NAD(P)H oxidase (Nox), and response to cytokine and growth factor receptors) and some metabolic enzymes as side products (so-called endogenous sources) [70]. The second kind of ROS generation source exogenous includes physical and chemical factors such as ultraviolet light, ionizing radiation, pollutants, pathogens, medicaments, chemotherapy, and lifestyle [67, 68]. Examples of ROS and RNS include superoxide anion radical (O_2_^·−^), hydroxyl radical (HO^·^), hydroperoxyl radical (HO_2_^·^), alkoxyl radical (RO^·^), peroxyl radical (ROO^·^), hydrogen peroxide (H_2_O_2_), singlet oxygen (^1^O_2_), nitric oxide (NO^·^), and peroxynitrite (ONOO^−^) [69]. Evidence shows NAD(P)H oxidase complexes as generators of ROS “have specific subcellular localization” producing these species in specific cellular compartments [71]. In study on human cell line MOLM-13 (acute myeloid leukemia), Guida et al. [71] reported that 1, 2, and 4 isoforms of NOx (Nox1, Nox2, Nox4, respectively), p22phox, and Rac1 gene subunits were expressed in cell lines of myelodysplastic syndrome/acute myeloid leukemia samples with damaged DNA in the nuclear fractions. The authors also found that Nox4 isoform was localized in the nucleus and inhibition of the isoform activity was followed by decreased formation of nuclear ROS in the nucleus. Further, the authors maintain that Nox4 isoform can participate in the regulation of information transfer from DNA to a messenger RNA (mRNA) due to ROS formation in the specific nuclear domains. In addition, it has been reported that Nox4 interacts with the Akt and ERK (extracellular signal-regulated kinase) signal transduction pathways. Based on available information, many transcription factors, for example, nuclear factor-*κ*B (NF-*κ*B), activator protein 1 (AP-1), a basic tumor suppressor gene (p53), NF-E2-related factor (Nrf2), and hypoxia-inducible factor-1*α* (HIF-1*α*), exhibit the redox sensitivity; the formation of ROS in the nucleus linked with Nox4 activity influences the nucleus redox homeostasis, thus regulating transcription of the redox-sensitive factors, gene expression and regulation, affecting cell growth, differentiation, senescence, and apoptosis [71, 72]. Findings on the role of Nox4 in nuclear ROS production highlight the molecular mechanisms (at the level of the cell nucleus) responsible for genomic instability in myelodysplastic syndromes [73]. The knowledge of OS regulation in cells is important in both the cancer development and anticancer therapies [74].

Evidence has shown the carcinogenesis process is characterized by hypoxia and inflammation [75–79]. Metabolism of normal cells undergoes change during their transformation to cancer cells, i.e., the phospholipid peroxidation pathway is shifted towards fermentative glycolysis, a process known in the subject literature as “Wartburg effect” or the Myc/hypoxia-induced metabolic pathway [80, 81]. This process results from the uncontrolled growth signaling, deregulation of the family of regulator genes and protooncogenes (c-Myc), and hypoxia-inducible factor 1 (HIF-1) activities. In this way, the glycolytic enzymes are induced, and the final product of glycolysis pyruvate in mitochondria is reduced [82]. In tumors, oncogenes and tumor suppressor agents activate mitogenic pathways such as Ras-Raf-ERK (extracellular-signal-regulated kinase) and P13K- (phosphatidylinositol 3-kinase-) AKT (protein kinase B) pathways which promote glucose metabolism; cell growth, proliferation, and survival; and apoptosis [81]. In cancer cells, these pathways are activated by Myc and HIF-1 promoting metabolism and accumulation of its products. Thus, cancer cells can respond to a stressor, i.e., OS via increased activation transcription factor (ATF)-4 [83] and nuclear factor erythroid 2-related factor (Nrf2), a transcription activator responding to ROS and electrophile generation and promote their detoxification being procarcinogenic in neoplasia expression [84]. Recently, the current state of knowledge on “Wartburg effect” and experimental data on the glycolysis genetic disruption and novel cell death mechanisms, based on the responses of cancer cell lines, with potential utility for anticancer strategy, have been reported by Ždralević et al. [81]. The authors showed that, in contrast to normal cells which derive their energy from respiration, the suppression of “Wartburg effect” has little effect on proliferation of cancer cells and tumor growth because of efficient reactivation of respiration as a source of energy and increased redox status. As cancer cells in this state are strongly susceptible to glutathione depletion [85], and cysteine/glutamate antiporter xCT controls glutathione synthesis and Nrf2 contributes to respiratory re-activation, Ždralević et al. proposed a mechanism of cancer cell death called “ferroptosis” as a new target for cancer therapy, i.e., an inhibition of xCT antiporter; this allows for the use of the acute lethal peroxidation of phospholipid to cause the death of aggressive cancer cells [81].

Among the major cellular growth- and proliferation-linked signal transduction pathways involved in responding to OS is the mitogen-activated protein kinase (MAPK) cascade. Cells' response to stress involves several mechanisms ranging from the stimulation of pathways promoting survival to the initiation of cell death and possible elimination of damaged cells; this process depends on the type of cell as well as the nature and duration of the stress [86]. In the last two decades, the apoptosis signal-regulating kinase (ASK) family members ASK1, ASK2 and ASK3, the key molecules in MAPK signal cascade, have attracted much attention. ASK1 participates in the regulation of cell survival, proliferation, inflammation, and apoptosis [87]. Evidence has shown that ASK1 in response to OS and the bacterial component lipopolysaccharide (LPS) activates c-Jun N-terminal kinase (JNK) and p38 kinase which are involved in apoptosis and inflammation in response to stressful stimuli, where ROS are the stress mediators and control many cellular processes [87, 88]. The finding of Matsuzawa showed that the ASK1-p38 signaling pathway is critical for the formation of inflammatory cytokines (TNF-*α*, IL-6, IL-12, and IL-1*β*); these finding demonstrate ASK1 signaling is involved in mammalian innate immunity [88]. Importantly, ASK1 activation is regulated by thioredoxin (Trx), an antioxidant that in a reduced form contains two thiol (SH) groups playing the basic role in the regulation of redox signaling [89]. In an inactive state, ASK1 forms inactive molecular complex with reduced Trx. Under conditions of OS, the reduced form of Trx undergoes oxidation followed by the complex dissociation, leading to the conversion of OS signal to a phosphorylation-dependent signal; thus, Trx acts as an efficient antioxidant. Further, the recruitment of the tumor necrosis factor-*α* receptor-associated factors (TRAFs) 2 and 6 activate an inactivate ASK1 molecule to its active form. Matsuzawa underlines that activation of the TRAF6-ASK1-p38 pathway dependent on ROS level is needed for the generation of cytokines and phagocytosis during the innate immune system response to stress. Research findings have shown that ASK1-p38 signaling induces various immune diseases such as multiple sclerosis, rheumatoid arthritis, and cardiovascular and various infectious diseases. For the comprehensive reviews and extensive discussion on innate immune system response to stress, References [87, 88, 90] are illustrative.

The next important consequence of excess ROS is aging, the process which touches all living organisms. There is abundant research confirming association between severe OS stress and elevated risk of age-related diseases. Aging is considered the universal multifactorial and progressive process accompanied by loss of tissue and organ function over time [91–93]. Among many theories of aging are the free radical theory and mitochondrial theory [92, 94, 95]; both theories hypothesize that ROS are one of the main reasons of aging. According to the free radical hypothesis of aging, this process is caused by the accumulation of oxidation products of lipids and proteins and structural damages in mitochondrial DNA, RNA molecules by ROS, and RNS. In addition, cellular antioxidant systems are weakened, and the intracellular level of ROS is increased due to increased rate of formation. The aging process can be associated with mutations and neoplastic transformation [68, 96]. Several transcription factors are modulated by ROS, e.g., NF-*κ*B, AP-1, Nrf2, p53, hypoxia-inducible factor-1*α* (HIF-1*α*), signal transducer, and activator of transcription 3 (STAT 3) [68].

It is maintained that ROS participates in the initiation and progression of cell aging, and their accumulation can cause OS followed by mRNA damage, a decrease in mitochondrial function, and progression of age-related diseases (i.e., arthritis, diabetes, vascular diseases, dementia, and cancer) [77, 97]. (For a detailed review on the role of OS in aging and age-related diseases, see reviews of Liguori et al. [92] and Davalli et al. [95].) Further, the aging process is characterized by chronic low-grade systemic inflammation caused by limited elimination of damaged cells and biomolecules [96].

The decreasing potency of endogenous antioxidant systems with growing age increases susceptibility of cell and tissues to OS [98]. Evidence shows transcriptional activities of IL-1*β*, IL-6, TNF-*α*, cyclooxygenase-2 (COX-2), nitric oxide synthase (iNOS), and several other proinflammatory proteins are increased during aging. Further, aging is also characterized by the increased number of monocytes and neutrophils, and C-reactive protein (CRP) levels. In turn, more recent evidence has also shown that depletion of Nrf2 activity is involved in the development of age-related diseases [99]. For additional review, see [98]. Comprehensive experimental studies over the past decade have shown OS can impair stem cells' redox homeostasis in hematopoietic, neural, and muscle stem cell compartments [100]. Stem cells are involved in preserving tissue homeostasis and the repair of damaged DNA through replacing damaged/lost cells; thus, they play an important role for functional tissue and organ maintenance [101]. Therefore, the regulation of balance between quiescence, self-renewal, and differentiation is crucial for stem cell functioning during early development and cell homeostasis [100–102]. The cellular changes linked with aging (called cellular senescence) may lead to irreversible arrest of cell growth and changes in the cellular phenotype owing to the impaired signaling [103].

The response mechanism of stem cells on OS is not well recognized, but accelerated telomere shortening, increased double-stranded DNA breaks, and nuclear A-type prelamin accumulation, followed by stem cell senescence or premature aging and cell death, have been reported [103]. An important role of A-type prelamins, i.e., the proteins responsible for the structural and functional integrity of the cell nucleus, is the ability to modulate intracellular redox homeostasis [101]. The experimental study of aging processes during *in vitro* expansion of stem cells from amniotic fluids (hAFSCs cultures) by Casciaro et al. showed low levels of intracellular ROS prevented nuclear A-type prelamin accumulation and Nox4 nuclear activity towards ROS production [101]. This finding supports a key role of OS in the cellular aging process. In addition, Porto et al. explored the role of ROS in bone marrow hematopoietic stem cell (HSC) aging finding the aged mice cells (24 month aged) had increased intracellular concentrations of the following compounds: O_2_^·−^ (1.4-fold), H_2_O_2_ (2-fold), and NO (1.6-fold), peroxynitrite/OH^·^ (2.5-fold) comparing with young mice cells (2 months old) [104]. Moreover, the authors observed a 3.3.-fold increase in the number of HSCs during the lifetime of aged mice comparing with the cell number of young mouse cells and found a positive correlation between cell aging and age of mice: 12-month-old mice had a 4.2-fold increase in DNA damage and a 2-fold in apoptosis, while 24-month-old mice 6-fold and 4-fold increases, respectively. In addition, the older mice had a shorter telomere length compared with the middle-aged mice.

Due to the repair property of stem cells and their potential application as promising drug targets for several pathology in regenerative medicine, the current research has focused on the role of Nox isoforms in stem cell differentiation, renewal, and cancer stem cell growth and survival [105, 106] and Nox inhibitors to regulate OS in stem cells [102, 107]. Until now, evidence showed Nox isoforms play a leading role in all the abovementioned processes. Several of the seven isoforms participate in each process, Nox4 plays a crucial role in O_2_^·−^ and H_2_O_2_ generation, and the Nox2 and Nox4 activities were found to be increased in majority of stem cell types [105]. There are also reports that proliferation of some solid cancers and their resistance to chemotherapy are dependent on tumor-initiating cells of cancer stem cells; the cells exhibit the self-renewal ability and potency to develop tumor and metastasis [106]. Although the pathologically altered expression and activity of stem cells in response to OS are linked with several inflammatory diseases including the development of several types of cancers (e.g., lung, colon, and prostate) and their progression, the mechanisms of Nox-formed ROS in carcinogenesis are not fully understood.

A number of studies indicated that an excess of ROS/RNS followed by OS, i.e., a disturbance of equilibrium between prooxidant processes and the protective strategies of antioxidant defense system, is genotoxic and can induce DNA damage; thus, they may play a key role in cancer development and progression [75, 77, 78] and in neuropsychiatric disorders, such as schizophrenia or depression [108]. Excess intracellular ROS may result in neuronal membrane damage, alter a broad range of its functions, and cause multineurotransmitters' pathologies [69].

Long-lasting permanent stress can lead to upregulation of the signal transduction in cancer cells and the tumor environment followed by tumor growth and progression [109]. Shin et al. [6] suggested individuals who experienced long-lasting permanent stress can accumulate cellular damage followed by their transformation into cancer cells. Interestingly, there is evidence that women who experience traumatic stress have shortened telomeres becoming 10 years older compared to low-stress women [110, 111]. This means the region of repetitive DNA of a chromosome is less protected from deterioration. Accelerated rate of telomeres' degradation is associated not only with aging but may also lead to oncogenesis of somatic cells [112]. The implications of OS and other biological processes, such as accumulation of DNA damage, telomere shortening, elevated levels of proinflammatory cytokines, impaired immune system, inflammation, and deregulated levels of stress hormones, are suggested to occur in the psychological disorders and in cancer diseases [16, 18]. OS has been shown to play an important role in tumor initiation, promotion, and progression [67, 68] and is implicated in the pathogenesis of various biological systems and organs (reviewed lately by Kruk et al. [77]). The role of PS in increased DNA damage has been supported by human studies, in vitro studies with animals, and cell lines exposed to stress hormones [14, 59, 113]. These multivariate complexities show the difficulty in determining and expressing the association between PS and cancer risk.

### 4.1. Limitations

There are several limitations in this review. First, our conclusions are based to a large extent of reviewed studies on case-control, 11 of 26 observational studies, where selection bias, recall bias, and detection bias are common. Individuals with cancer could have a higher level of PS perception as a factor for cancer than healthy individuals, thus, leading to the over-reporting of stress exposure. Second, selection of information from retrieved articles might be subjective and also lead to bias. In order to minimize potential selection bias, two research staff members independently selected articles for inclusion. Third, we analyzed only studies that met our inclusion criteria and excluded other studies. Finally, we included only articles in English in this overview.

## 5. Conclusions

The result of this overview demonstrates that review studies differ in their assessment of the contribution of behavioral factors such as chronic stress, severe life events, and personality to onset of cancer. The research evidence points to a prominent role for chronic daily life event stress, severe life events, depression, and social isolation in cancer growth and metastasis. Results from recently published observational epidemiologic studies, being the main subject of this review, have continued to establish an evidence base suggesting psychosocial factors may be risk factors for specific types of cancer incidence. In addition, recent evidence on psychosocial stress, including job stress, and specific types of cancer other than BC association is stronger than previously suggested in the literature. This confirms the conclusion of an important role for chronic stress in aging, development of cancer, and cancer growth and metastasis, based on previous studies analyzed in the review papers. However, several discrepancies were found in the literature, which include methodological differences in the measurements of PS type, timing, limited number of prospective studies, small sample sizes, and a lack of adjustments for the main risk factors for specific types of cancer and lifestyle factors. The effect of chronic PS on carcinogenesis has a plausible biological rationale. The body of evidence indicates unregulated release of CATs (adrenaline, noradrenaline) by the activation of HPA axis, deregulation of SNS, and adrenal glands in response to PS increases the generation of proinflammatory proteins and disturbs cell homeostasis involving OS along with a decrease in cellular immunity. Evidence shows the important role of Nox isoform inhibitors in regulation of OS in stem cells particularly ASK1 in innate immune signaling, highlighting their importance in the cellular aging and cancer. As understanding these processes grow, there is hope they may offer new clues for therapeutic approaches in treating cancer. Our findings may become a greater part of health prevention strategy associated with modifiable behavioral factors, e.g., work conditions or coping with self-perceived stress. Finally, this article may inspire or guide future studies on the PS-cancer risk association of high methodological quality.

### 5.1. Future Perspectives

Determining the causal relationship between PS and cancer risk requires large-scale research efforts involving participation from multidisciplinary health specialists. This is due to the multivariate complexities associated with sources of PS, the relationship between stress and cancer disease, and the complicated nature of modifiable behaviors. Future epidemiological observational studies require larger study populations, international collaboration, unselected patient groups, and questionnaires providing detailed behavioral information to assess possible risk factors for particular cancer types considered as potential confounders. In addition, future studies should provide a significant measure of PS regarding the type and duration using valid and reliable scales to ensure a clear dose-response relationship. This approach could clarify existing biological mechanisms and identify additional risk pathways including ROS-induced associated with the relationship between PS and particular cancer types' outcome as well as the role of ROS/RNS in aging.

## Figures and Tables

**Table 1:** Characteristics of the most relevant epidemiological studies on psychosocial factors and risk for breast cancer.

First author/reference/location	Study design/sample	Follow-up years	Exposure	Effect size (95% CI)	Control for confounding
Kruk, 2012 [23] Poland	Hospital-based case-control study 858 cases, 1085 controls	January 2003-May 2007	Death of a close family member	OR = 2.48 (1.70-3.64)	Age, lifetime recreational physical activity, place of residence, education, age at menarche, breastfeeding, family history of breast cancer, exposure to cigarette smoke, alcohol consumption, and intake of vegetables and fruits
			Personal injury, illness	OR = 2.60 (1.63-4.62)	
			Imprisonment, trouble with law	OR = 2.94 (1.56-5.45)	
			Retirement	OR = 1.52 (1.08-2.45)	
			Lifetime scores > 210	OR = 5.09 (3,41-8,50)	

Wang et al. 2013 [24] Taiwan	Case-control study (157 cases, 314 controls)	June 2009-June 2011	High perceived PS	OR = 1.65 (1.10-2.47)	Adjusted for potential lifestyle factors
			Joint interactions high perceived PS with the following:		
			Alcohol intake ≥ 11.0 g/day	OR = 2.91 (1.23-6.86)	
			Smoking ≥ one cigarette/day	OR = 2.52 (1.16-5.47)	
			Low physical activity	OR = 3.36 (1.77-6.36)	
			High fried and stir-fried food	OR = 3.18 (1.79-5.63)	
			High meat and sea food intake	OR = 1.89 (1.09-3.27)	

Li et al. 2016 [25] China	Comparative case-control study 582 cases, 540 controls	May 2013-May 2015	Frequent depression	OR = 1.32 (1.00-1.75)	Adjusted for main risk factors
			Negative emotional experiences	OR = 1.15 (1.03-1.29)	
			Disharmonious marital status	OR = 1.16 (1.06-1.26)	

Sawada et al. 2016 [26] Japan	Prospective study 29,098 women, 209 cases identified	21 years	Psychological traits:		Age, study area, education, family history of breast cancer, age at menarche, age at menopause, parity, use of exogenous female hormones, alcohol intake, daily walking, exercise, sedentary work, height, and BMI
			“Ikigai” agree strongly	HR = 0.81 (0.41-1.62)	
			Decisiveness agree	HR = 1.07 (0.62-1.85)	
			Ease of anger agree	HR = 0.98 (0.50-1.76)	
			Perceived stress agrees strongly	HR = 1.00 (0.56-1.78)	

Schoemaker et al., 2016 [27] UK	Prospective cohort study 106,000 women, 1783 cases	2003-July 2012	Breast		Age, age at menarche, age at first birth, parity breastfeeding, hormone use, BMI, smoking, alcohol intake, physical activity, family history of BC, and socioeconomic status
			Death of close relatives	RR = 0.87 (0.78-0.97)	
			Death of husband/partner	RR = 1.13 (0.88-1.46)	
			Divorce/separation	RR = 1.15 (0.96-1.38)	
			Personal illness/injury	RR = 1.03 (0.87-1.22)	
			Mather death	RR = 1.31 (1.02-1.67)	

Yeh and Lee 2016 [28] Taiwan	Prospective cross-sectional study, 54 cases, 1106 controls	On day prior mammography	Borderline anxiety *vs* no anxiety	OR = 3.099 (1.685-5.698)	These risks were adjusted only for educational factors. Results of multiple logistic regression models presented goodness-of-fittest *P* < 0.05
			Anxiety *vs* no anxiety	OR = 2.173 (1.009-4.684)	
			Depression *vs* no depression	OR = 4.497 (1.643-12.308)	
			Stress	OR = 1.124 (1.062-1.190)	

Özkan et al. 2017 [29] Turkey	Hospital based case-control study 491 cases, 512 controls	September 2013-September 2014	Experience of stressors in the last 5 years	OR = 5.662 (3.767-8.511)	Age, BMI, family history of cancer, marital status, employment status, and economic conditions
			Insufficient social support perception	OR = 2.166 (1.371-3.424)	
			Avoidance coping strategy	OR = 1.882 (1.271-2.785)	
			Child trauma	OR = 1.48 (1.14-1.91)	
			Major life events	OR = 1.76 (1.22-2.53)	
			Chronic stress	OR = 2.01 (1.52-2.67)	

Butow et al. 2018 [30] Australia	Prospective cohort study (2,739 women, 103 cases)	May 2001-December 2010 (mean follow-up 7.2 years)	Experience of stressors in the last 3 years: severe (death of a spouse/child, handicapped child requiring full-time care) moderate (e.g., buying/selling a home)	Lack of statistical significance in all unadjusted and adjusted models	Family history of cancer, age at menarche, ovarian cancer, physical activity, HRT use, oral contraception use, BMI, anxiety, depression, parity, smoking, and number of live births

Fischer et al. 2018 [31] USA	Case-control study (664 cases, 203 population-based controls)	March 1st 1994	Cumulative adverse life events	OR = 1.63 (1.00-2.66) *P* trend = 0.045	Adjusted for age, age at first full-term pregnancy, menopausal status, family history of BC, HRT use, smoking, race/ethnicity, education level, and physical activity
		February 28 1995	Previous personal illness: Perceived as stressful	OR = 2.84 (1.96-4.11)	
			Perceived as non-stressful	OR = 3.47 (1.34-8.94)	

Yildirim et al. 2018 [32] Italy	Hospital-based case-control study (250 cases, 250 controls)	September 2013	Loss of father during childhood	OR = 2.68 (1.30-5.52)	Adjusted for age, education status, marital status, work status, economic status, social security, age at menarche, age at first pregnancy and birth, family history of cancer, and history of psychiatric disorder
		September 2014	Serious stressor within the last five years	OR = 4.72 (3.18-7.03)	
			Inadequate social support	OR = 1.83 (1.23-2.73)	

Abbreviations: HR: hazard ratio; OR: odds ratio; RR: relative risk; CI: confidence interval; BMI: body mass index; PS; psychological stress; HRT: hormone replacement therapy.

**Table 2:** Characteristics of the most relevant epidemiological studies on psychosocial factors and risk for cancer other than breast cancer.

First author/reference/location	Study design/sample	Follow-up years	Specific cancer site/type of measurement	Effect size (95% CI)	Control for confounding
Cabaniols et al. 2011 [34] France	Case-control study (122 cases, 122 controls)	January–December 2005	Brain		Age, sex
			Major life events	OR = 1.90 (1.13-3.20)	
			Daily stress	OR = 0.90 (0.49-1.71)	
			Work	OR = 0.69 (0.27-1.74)	

Huang et al. 2013 [35] Sweden	Nested case-control study (16,522 cases, 82,107 controls)	1991-2009	Pancreatic cancer Psychological stress induced by		Age, sex, education, socioeconomic status region of residence, total number of children
			the death of a child	OR = 1.27 (1.12-1.45)	
			or loss of a child due to suicide	OR = 1.23 (1.03-1.146)	
			Persons with a history of psychiatric illness after child loss	OR = 1.43 (1.17-1.76)	

Vasunilashorn et al. 2013 [36] Taiwan	Prospective cohort study (9,302 adults)	1999-2010	All diseases Self-reported perceived stress (health, financial situation, occupation, relation with family members, marriage)	Mortality risk HR = 1.19 (1.13-1.26)	Age, education, marital status, survey wave, tobacco smoking, alcohol intake, sex, mobility limitations

Momen et al. 2013 [37] Denmark and Sweden	Nationwide follow-up cohort study (2,729,308 children born in Denmark 1968-2007; 3,395,166 born between 1973 and 2006 in Sweden) 1,505,938 children experienced bereavement, and 9,823 were diagnosed with cancer before the age of 15 years	Started from birth and ended at date of cancer diagnosis, death, emigration, day before 15^th^ birthday or at 2007 in Denmark, and 2006 in Sweden	All childhood cancers	HR = 1.10 (1.04-1.17)	Country, maternal characteristic at birth whether child was a twin
			Central nervous system cancer	HR = 1.14 (1.02-1.28)	
			Leukemia	HR = 1.12 (1.00-1.26)	
	

Azizi & Esmaeili 2015 [38] Iran	Case-control study (207 cases, 207 controls)	April 2013–March 2014	Colorectal cancer Death of dears/child, parents, spouse, and first-degree families	OR = 2.49 (1.41-5.13)	Age, sex, family history of colorectal cancer, history of diabetes, smoking, BMI, physical activity

Kikuchi et al. 2017 [39] Japan	Prospective cohort study 61,563 participants (25,018 men; 36,545 women) 330 rectal cancer cases, 680 colon cancer cases	Maximum 21 years (mean 13 years)	Rectal cancer Daily life stress Moderate level:		Age, BMI, family history of colorectal cancer, smoking habit, alcohol drinking, sleep duration/night, frequency of green leafy vegetables intake, daily time walking, bowel movement frequency, age of graduation, marital status, employment status, the number of children
			men women	HR = 2.16 (1.23-3.78) HR = 3.20 (1.46-7.03)	
			High/severe level:		
			men women	HR = 1.75 (1.14-2.69) HR = 1.83 (1.01-3.31)	
			Colon cancer No statistically significant association		

Blanc-Lapierre et al. 2017 [40] Canada	Population-based case-control study (3,103 cases, 512 controls)	1979-1985	Different types of cancer, workplace PS		Age, ethnicity education, family income, respondent status, site specific, nonoccupational and occupational covariates like smoking, occupational exposure to asbestos and silica, BMI, exposure to aromatic amines, smoking, alcohol intake
			Lung	OR = 1.33 (1.01-1.75)	
			Colon	OR = 1.51 (1.15-1.98)	
			Bladder	OR = 1.37 (1.03-1.81)	
			Rectal	OR = 1.52 (1.10-2.10)	
			Stomach	OR = 1.53 (1.08-2.15)	

Kim et al. 2017 [41] Korea	229 cancer patients among them 77 with PD	November 2009–March 2011	Gastric Psychological distress	Disease stages I-III, 5-year DFS rate 60% *vs* 76% Disease stage IV Median OS 12.2 *vs* 13.8 months. DFS and OS were estimated compared with patients without PD HR = 2.47 (1.07-5.68)	Age, gender, Eastern Cooperative Oncology Group performance status (0-3; 4), marriage, education, employment, and adjuvant chemotherapy

Chang et al. 2015 [42] Korea	Prospective study 601,775 people (502,297 men, 99,478 women) with depression Cases: Men 49,744; women 7,860	20 years	Prostate cancer Minor depression	HR = 1.13 (1.05-1.23)	Age, smoking, alcohol intake, exercise, BMI, cholesterol, blood sugar, hypertension, cancer family history
			Cervical cancer		
			Major depression (women)	HR = 0.90 (0.83-0.98)	
			Overall cancer Major depression:		
			men women	HR = 1.05 (1.01-1.09) HR = 0.90 (0.83-0.98)	

O'Neil et al. 2014 [43] International	19 World Mental Health surveys with DSM-IV (*n* = 52,095), 1,499 cases	10	Overall self-reported cancer diagnosis DSM-IV		Age, gender, person-year, country, alcohol consumption, country
			One disorder	OR = 1.3 (1.1-1.6)	
			Three disorders	OR = 1.6 (1.2-2.2)	
			> Five disorders	OR = 2.3 (1.6-3.3)	

Archer et al. 2015 [44] UK	Prospective cohort study (*n* = 6,983), 776 cases	17.4	Overall cancers Chronic depressive symptoms	HR = 1.03 (0.71-1.49)	Age, gender, employment grade, smoking, alcohol intake, meat consumption, physical activity, BMI, systolic blood pressure, respiratory illness, longstanding illness
	285 cases	<9	New depressive symptoms	HR = 1.89 (1.23-2.90)	

Li et al. 2014 [45] China	Case-control study (250 cases, 500 controls)	January 2007–July 2013	Prostate cancer		Smoking, alcohol intake, physical activity, marital status, red meat consumption, tea consumption, urinary system diseases, family history of BC, vegetables consumption
			Occupational setback	OR = 1.61 (1.00-2.59)	
			Marital separation	OR = 1.94 (1.29-1.91)	
			Self-contained suffering	OR = 2.37 (1.58-3.55)	
			High sensitivity to the personal comments	OR = 1.73 (1.18-2.54)	

Song et al. 2017 [46] Japan	Prospective study 101,708 participants declaring perceived stress at baseline, 17,161 cancer cases	5 years and 10 years	Overall cancer Perceived stress at baseline	No association between baseline PS level and cancer risk. Slightly (4-6%) elevated HR in the group declaring higher PS levels *vs.* low-stress group. For long-term PS (79,301 participants, 963 cancer cases) with always a high PS level: HR = 1.11 (1.01-1.22) in all group and HR = 1.19 (1.05-1.34) in a subgroup of men *vs*. subjects declaring always a low level of PS.	BMI smoking status, alcohol consumption, fruit/vegetable intake, physical activity, living arrangement, occupation, family history of cancer, study area.

Blank-Lapierre et al. 2017 [47] Canada	Hospital based case-control study (1,933 cases, 1,994 controls)	2005-2009	Prostate cancer Men aged <75 years at diagnosis		Age, ancestry, first-degree family history of PCa, family income, education. Marital status, BMI, type 2 diabetes, depression treated with medication, alcohol intake, smoking, physical activity at work, frequency of fruit, and vegetable consumption
			Exposure to job stress duration > 30 years	OR = 1.40 (1.07-1.82)	
			Low-grade PCa cases Exposure to job stress > 30 years	OR = 1.36 (1.02-1.08)	
			High-grade PCa cases Exposure to job stress > 30 years	OR = 1.53 (1.03-2.29)	
			Perceived workplace stress duration linkage with a higher PCa risk	OR = 1.12 (1.04-1.20) per 10-year increase	

Vesterlund et al. 2017 [48] Denmark	Danish Nurse Cohort, 6571 participants (854 cases)	January 2000–December 2013	Job strain high *vs* low		Age, night shifts and full-work time, smoking, alcohol, BMI, physical activity at work and leisure time
			Overall cancer	HR = 0.84 (0.7-1.1)	
			Hormone-related cancer	HR = 0.82 (0.6-1.2)	
			Virus immune-related cancer	HR = 1.08 (0.5-2.5)	
			Digestive cancer	HR = 0.66 (0.3-1.3)	
			Lung cancer	HR = 1.05 (0.5-2.1)	

Jafri et al. 2019 [49] USA	Matched case-control study (102 cases, 199 controls)	May 2015–December 2016	Lung Stressful life events, past 5 years	OR = 2.21 (1.11-4.37)	Smoking, family history of lung cancer

Abbreviations: HR: hazard ratio; OR: odds ratio; RR: relative risk; DFS: disease-free survival; PD: psychological distress; OS: overall survival; PCa: prostate cancer; RR: relative risk; CI: confidence interval; BMI: body mass index; DSM-IV: Diagnostic and Statistical Manual of Mental Disorders.
